# Introduction of F446I mutation in the K13 propeller gene leads to increased ring survival rates in *Plasmodium falciparum* isolates

**DOI:** 10.1186/s12936-018-2396-0

**Published:** 2018-07-05

**Authors:** Jing Wang, Yufu Huang, Yuemeng Zhao, Run Ye, Dongmei Zhang, Weiqing Pan

**Affiliations:** 1 Department of Tropical Infectious Diseases, Second Military Medical University, Shanghai, 200433 China; 2 Institute for Infectious Diseases and Vaccine Development, Tongji University School of Medicine, 1239 Siping Road, Shanghai, 200092 China

**Keywords:** *Plasmodium falciparum*, K13 propeller gene, F446I mutation, Artemisinin resistance

## Abstract

**Background:**

Mutations in the *Plasmodium falciparum k13* gene are associated with artemisinin (ART) resistance. However, it is unclear whether the F446I mutation, the most prevalent allele at the China–Myanmar border and north of Myanmar, is associated with ART resistance. Therefore, the aim of this study was to investigate the role of this mutation in ART resistance by generating transgenic parasites expressing the F446I mutant allele.

**Methods:**

The transgenic parasites carrying the F446I or C580Y mutation in both 3D7 and FCC1/HN isolates were generated by single crossing-over recombination and verified using PCR and gene sequencing. The ring-stage survival assay of 0–3 h (RSA_0–3 h_) was used to evaluate ART susceptibility of the transgenic parasites in vitro.

**Results:**

Four transgenic parasite lines named 3D7^F446I mut^, 3D7^C580Y mut^, FCC1/HN^F446I mut^ and FCC1/HN^C580Y mut^ were successfully generated. These parasite lines showed no changes in the expression level of *k13* when compared with their parent parasite isolates. However, introduction of the F446I mutation in *k13* of the 3D7 and FCC1/HN isolates led to elevated ring survival rates detected using RSA_0–3 h_ when subjected to both 700 and 20 nM concentrations of dihydroartemisinin. The survival rates were similar to those detected in the parasite lines with the C580Y mutation.

**Conclusions:**

Insertion of the F446I mutation in *k13* led to increased ring survival, suggesting that this mutation may be associated with ART resistance and could be used as a molecular marker for monitoring ART-resistant parasites. The results also highlights the importance of surveillance of F446I mutants for containing the resistant parasite.

## Background

Artemisinin (ART) is a quick, effective anti-malarial drug that was discovered in China [1, 2]. Since ART-based combination therapies were recommended by the World Health Organization (WHO) as a first-line treatment of malaria in 2005, the global malaria burden has significantly declined [3]. However, in 2008, ART resistance was reported in *Plasmodium falciparum* parasites in western Cambodia; a delay in clearance time was observed in infected patients who had received the standard 3-day treatment [4, 5]. The resistance has spread across the mainland of Southeast Asia, including Cambodia, Thailand, Vietnam, Myanmar, and China–Myanmar border areas [6, 7]. The spread of ART resistance, particularly in Africa, will certainly threaten global malaria control programmes. Therefore, efficient tools, such as molecular markers, for the detection and surveillance of ART-resistant parasites are urgently required to contain the spread of the resistance.

Currently, tools available for the detection and surveillance of ART-resistant malaria parasites include measurement of in vivo parasite clearance rates and ring-stage survival assay of 0–3 h (RSA_0–3 h_) that is well correlated with in vivo parasite clearance rates [8]. The K13 propeller gene of *P. falciparum* (*k13*; PF3D7_1343700) has been identified as a molecular marker for monitoring ART-resistant parasites [9]. A single mutation after position 440 in *k13* is associated with slow parasite clearance rates in vivo and high parasite survival rates in vitro [7, 10]. The prevalent mutant alleles of *k13* in the Thai–Cambodian/Thai–Myanmar border areas are C580Y, Y493H, R539T, and I543T, while that in the northern Myanmar and China–Myanmar border areas is F446I [7, 9, 11]. Mutant alleles such as C580Y and R539T have been confirmed to be correlated to ART resistance characterized by long in vivo parasite clearance half-lives after ART treatment and high RSA_0–3 h_ survival rates [9, 12]. In addition, the association of the C580Y mutation with ART resistance was also confirmed through introduction of the mutation into *k13* [13]. However, F446I is the most prevalent mutant allele of *k13* at the China–Myanmar border and in northern Myanmar. Previous study reported that of 392 isolates from the China–Myanmar border, 128 had the F446I mutation with a prevalence of 32.7% [11]. Two other studies showed the F446I mutation with a prevalence of 36.5 and 27.2% in this area [14, 15]. It is unclear whether this mutation is associated with ART resistance. Therefore, homology recombinant technology was used to introduce the F446I mutation in *k13* gene of 2 distinct laboratory strains, 3D7 and FCC1/HN, of *P. falciparum* and investigate the impact of the mutation on ART susceptibility.

## Methods

### Parasite culture

The parasite culture was maintained in vitro in RPMI 1640 (Sigma-Aldrich, USA) with 10 mM HEPES (Invitrogen, California), 20 μg/ml hypoxanthine (Sigma-Aldrich, USA), 50 μg/ml gentamicin (Sigma-Aldrich, USA), 0.17% NaHCO_3_ (Sigma-Aldrich, USA), and 5 mg/ml AlbuMAXII (Gibco, USA). *Plasmodium falciparum* lines were propagated in human O^+^ erythrocytes (obtained from Changhai Hospital) and maintained at 37 °C in 5% CO_2_ and 90% N_2_. The parasites were synchronized using 5% d-sorbitol, as described previously [16]. Briefly, the cultured parasites were pelleted and treated with sorbitol at room temperature for 15 min and washed with non-serum RPMI1640 medium and maintained in standard culture medium.

### Construction of the pARL-*Pf*K13^F446I^ and pARL-*Pf*K13^C580Y^ recombinant plasmids

The pARL-GFP plasmid containing the drug screening gene hDHFR was used to construct the pARL-*Pf*K13^F446I^ and pARL-*Pf*K13^C580Y^ recombinant plasmid. The full-length *k13* gene with F446I mutation and 1563 bp of the C-terminal of *k13* gene was amplified using overlapping PCR. The primers for amplifying fragments with F446I were as follows: 1350 bp 5′-terminal fragment was amplified by primer pair of PARL-F (GCagatctATGGAAGGAGAAAAAGTAAAAACAAAAG) and K13-446-R (TCCACCTATACAAATTACTAATGGG); while 831 bp 3′-terminal fragment was amplified by primer pair of K13-446-F (CCCATTAGTAATTTGTATAGGTGGA) and PARL-R (GCggtaccTTATATATTTGCTATTAAAACGGAGTG). The two fragments were combined by PCR through overlapping sequences to generate the full length *k13* gene with F446I mutation by primer pair of PARL-F and PARL-R. Similar strategy was used to generate the1563 fragment of *k13* gene with C580Y mutation: the 1128 bp upstream fragment was amplified by primer pair of PARL-A-F (GCagatctAACGGAATTAAGTGATGCTAGTG) and K13-580-R (AGCAACATACATAGCTGATGA) while the 435 bp downstream fragment by K13-580-F (GCTATGTATGTTGCTTTTGAT) and PARL-R. The resulting fragments were cloned into the pARL-GFP plasmid by using the restriction sites *Bgl*II and *Kpn*I (Fermentas, USA) to generate recombinant plasmids of pARL-*Pf*K13^F446I^ and pARL-*Pf*K13^C580Y^. The recombinant plasmids were purified using the Endo Free Plasmid Maxi Kit (Qiagen, Germany).

### Parasite transfection

Synchronous *P. falciparum* 3D7 and FCC1/HN (isolated from Hainan Province and established for in vitro continuous cultivation in 1978) parasites were transfected with the recombinant plasmids as described previously [17]. Briefly, *P. falciparum* parasite culture with a high synchronous ring stage was prepared at 5% parasitaemia. 270 μl Cytomix buffer (120 mM KCl, 10 mM KH_2_PO_4_, 25 mM HEPES, 2 mM EGTA, 0.15 mM CaCl_2_, and 5 mM MgCl_2_) was mixed with 100 μg of the plasmid, and then with 50% haematocrit of the infected erythrocytes. The mixture was transferred to a 0.2-cm cuvette (Bio-Rad) and electroporated using the Bio-Rad MicroPulser with voltage 310 V and 950 μF. The transfected culture was selected using 2.5 nM WR99210 (Jacobus Pharmaceuticals) after 48 h until the WR99210-resistant parasites were obtained. DNA was extracted using QIAmp Mini kits (Qiagen, Germany) and detected by PCR using primers hDHFR, P3/P4, P4/P5, and P3/P6. The primers for identification of transfected parasites were as follows: hDHFR-F: TTAATGTATTATTCCAATGTGCATGAT, hDHFR-R: GTACTTAATGCCTTTCTCCTCCTG; P3: ACCATGATTACGCCAAGC; P4: AAAACGAACATTAAGCTGCC; P5: AACAAGGCGTAAATAT TCGTGT; P6: ATAAGAACATTTAAAATTTCTTCATT. The sequences of the modified *k13* gene were confirmed by DNA sequencing. RNA extracted from the F446I mutant parasite was digested by DNaseI (Sigma-Aldrich, USA) for 15 min in room temperature before transcribed to cDNA. The full-length of *k13* gene was amplified from the cDNA of F446I mutant parasite and subcloned to pEASY-T1 cloning Vector (purchased from Transgen Biotech, China). The primers for amplification were as follows: K13-F: ATGGAAGGAGAAAAAGTAAAAACAAAAG; K13-R: TTATATATTTGCTATTAAAACGGAGTG. The RNA of F446I mutant parasite after digestion was used as template to amplified *k13* gene as a no-RT control. The *k13* gene in the pEASY-T1-K13 recombinant plasmid was sequenced using the primers of M13 as follows: M13F-GTAAAACGACGGCCAGT; M13R-CAGGAAACAGCTATGAC.

### Antibody and western blot

The antibody against *k13* protein was produced by GenScript Corporation (USA). Briefly, a synthetic peptide (MEGEKVKTKANSISC) was conjugated with keyhole limpet haemocyanin and formulated with adjuvant. Rabbits were immunized for 4 times at 2-week intervals. The polyclonal antibodies were affinity-purified with peptides.

Western blot was performed as described previously [18]. Briefly, the parasite culture was pelleted and resuspended with 10 volumes of phosphate-buffered saline (PBS). Next, 3% saponin was added to lyse the erythrocytes at 0.03% final concentration on ice for 15 min, and the mixture was centrifuged. The pellets were washed 2 times with PBS and resuspended with a suitable volume of buffer A (20 mM HEPES, 10 mM KCl, 1.5 mM MgCl_2_, 1 mM EDTA, 1 mM EGTA, 50 mM NaCl, 0.65% NP-40, 1 mM DTT, and 1× protease inhibitor cocktail). The mixture was incubated for 1 h at 4 °C and centrifuged at 14,000 rpm to obtain the supernatant. The supernatant was separated by 10% SDS-PAGE gel and detected using *Pf*K13 polyclonal antibody (1:200). The Actin (Abcam-ab40854) was used as the loading control (1:1000). The secondary antibody was IRDye800CW goat anti-rabbit (LI-COR) diluted at 1:5000.

### RSA_0–3 h_ assay

RSA_0–3 h_ was performed as described previously [8]. Briefly, *P. falciparum* parasites were synchronized 2 times with 5% sorbitol to obtain tightly synchronous early-ring stage. The initial culture was prepared at 1% parasitaemia and 2% haematocrit, and then was exposed to 700 or 20 nM DHA (Sigma-Aldrich, USA) for 6 h. Then, the culture mixture was washed 3 times with non-serum 1640 culture medium, and cultured again for 66 h. The viable parasites were counted using a microscope in Giemsa-stained thin smears. The survival rate was calculated using *Ps*/*Pc* × 100%, where *Ps* is the parasitaemia in the DHA treatment group, while *Pc* is the parasitaemia in the control group. The counting was performed by two different experienced laboratories. Each assay was repeated for 3 times independently, with each group setting three technical repetitions. Each technical repetition was counted for 40,000 erythrocytes.

## Results

### Generation of constructs for transfection of the parasites

**Fig. 1:**
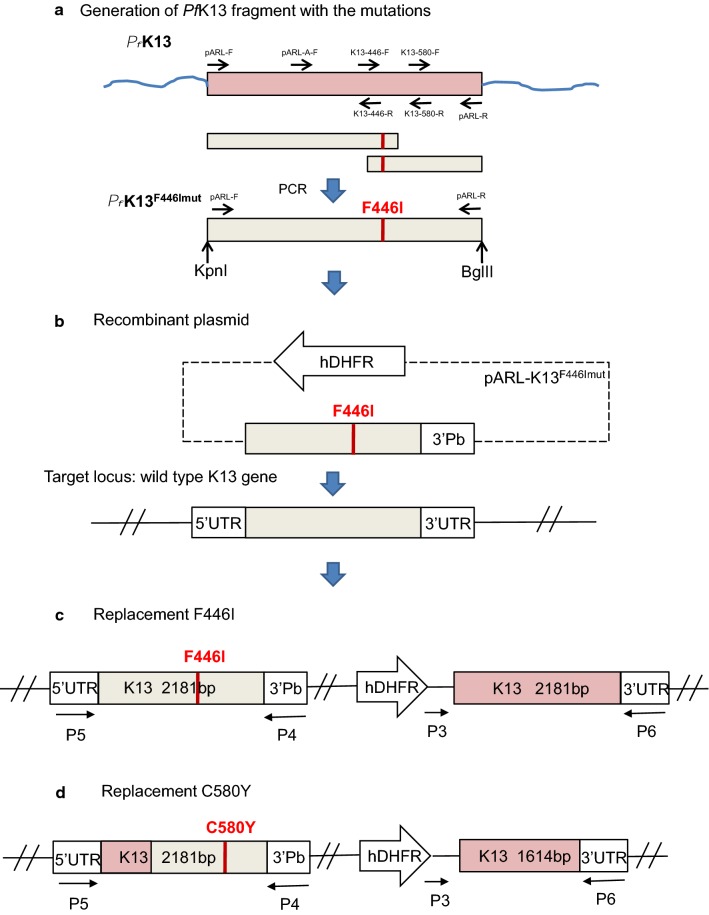
Strategy for generation of constructs for parasite transfection. **a** PCR amplification of *k13* gene with F446I mutation. **b** Insertion of *Pf*K13^F446I mut^ into the vector to generate the pARL-K13^F446I^ recombinant plasmid. **c** Integration of *Pf*K13^F446I mut^ into the target locus of the parasite chromosome. **d** Integration of *Pf*K13^C580Y mut^ into the target locus of the parasite chromosome

To generate the recombinant plasmids for the transfection, the fragments of *k13* gene were produced with either F446I or C580Y mutations by using PCR. For construction of the replacement F446I, 2 DNA fragments covering the entire *k13* gene (2181 bp) were amplified from the genomic DNA of *P. falciparum*. The two fragments were combined using PCR with an overlapping sequence of 15 bp in which a point mutation of T to A was introduced, resulting in changes to the corresponding amino acid residue from phenylalanine (F) to isoleucine (I) (designated as *Pf*K13^F446I mut^ gene). The full-length *Pf*K13^F446I mut^ gene contains the 5′ cleavage site of *Kpn*I and 3′ cleavage site of *Bgl*II, which allowed cloning of the entire gene into the vector pARL-GFP through corresponding sites in the vector and generation of the recombinant plasmid designated as pARL-K13^F446I^. The substitution of T to A in the *Pf*K13^F446I mut^ gene was verified by DNA sequencing. The overall strategy for the preparation of *Pf*K13^F446I mut^ is outlined in Fig. 1a.

A similar strategy was used for the generation of the *Pf*K13^C580Y mut^ gene. However, in this case, only a truncated *k13* gene (1563 bp) was amplified instead of the full gene. This is because of the position of the C580Y mutation near the C-termini and that only a partial sequence of the gene is needed for single crossing-over recombination. Similarly, two fragments covering the truncated *k13* gene were amplified and combined using PCR. A point mutation of G to A at nucleotide position 1739 was introduced through the overlapped neighbouring sequences of the 2 fragments, resulting in a change from cysteine (C) to tyrosine (Y) (designated as *Pf*K13^C580Y^ gene).

### Generation of transgenic parasites

Two distinct laboratory strains of *P. falciparum*, 3D7 (designated as 3D7^wt^) and FCC1/HN (FCC1/HN^wt^), were used for the transfection. Parasites in the high synchronous ring stage were transfected with individual recombinant plasmids by electroporation, as described above (Fig. 1b). The transfected culture was selected using 2.5 nM WR99210 at 48 h post-electroporation until the WR99210-resistant parasites were visible. Cloning of the resistant parasites was performed by limiting dilution. The resulting clones with the replacement F446I were designated as 3D7^F446I mut^ and FCC1/HN^F446I mut^ lines (Fig. 1c), and the clones with the replacement C580Y, as 3D7^C580Y mut^ and FCC1/HN^C580Y mut^ lines (Fig. 1d).

**Fig. 2:**
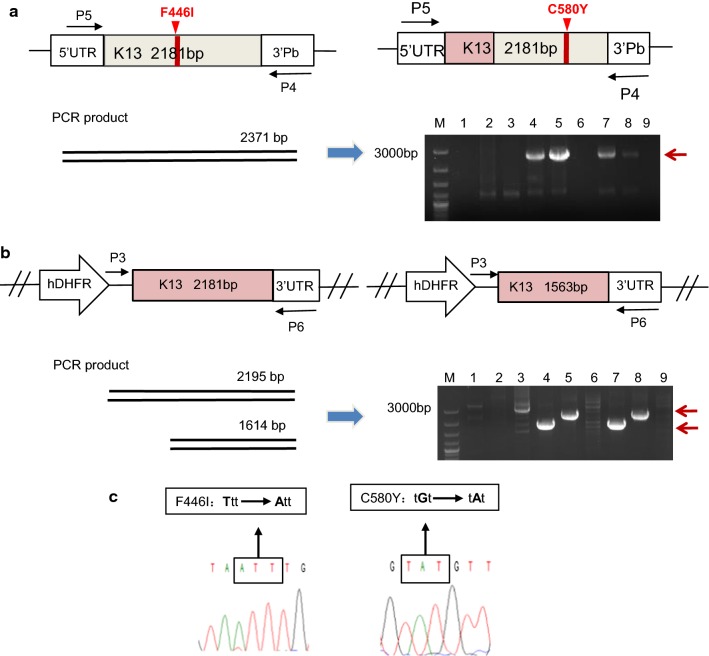
Verification of the integration of the F446I mutation into the target locus. **a** PCR amplification using the indicated primers produced the expected fragments in lanes 4, 5, 7, and 8 for the transgenic parasite lines, whereas no fragments were detected in the other lanes for the parent parasites. Lanes 1–9: negative control, pARL-K13^F446I^ plasmid, pARL-K13^C580Y^ plasmid, FCC1/HN^C580Y mut^, FCC1/HN^F446I mut^, FCC1/HN ^wt^, 3D7^C580Y mut^, 3D7^F446I mut^ and 3D7^wt^. **b** PCR analysis showed that the expected fragments were amplified from the transgenic parasites. Lanes 1–9, as indicated above. **c** Sequencing verification of the mutations from T to A for F446I and G to A for C580Y

The genomic DNA of each parasite clone was prepared for PCR verification of the integrations and mutant allele replacements. The P5 primer is located in 5′ UTR of *k13* in the chromosome while the P4 primer in 3′UTR of Pb DHFR-TS gene that is located in the plasmid. As shown in Fig. 2a, a 2371-bp fragment was amplified from the transgenic parasite lines with either F446I or C580Y mutation by using the P4/P5 primers, indicating that both *Pf*K13^F446I mut^ and *Pf*K13^C580Y mut^ were inserted into the target locus by single crossing-over recombination. No fragment was detected from their parent parasites, 3D7^wt^ and FCC1/HN^wt^ strains.

Similarly, the P6 primer is located in 3′ UTR of the gene in the chromosome, and the P3 primer, in the plasmid. Successful amplification of 2195- and 1614-bp fragments further demonstrated the correct integration into the loci of *Pf*K13 with F446I and C580Y mutations, respectively (Fig. 2b). In addition, the fragments amplified by the primer pair P4/P5 were sequenced, and the correct substitution of A for T for the F446I mutation and A for G for the C580Y mutation was confirmed (Fig. 2c).

**Fig. 3:**
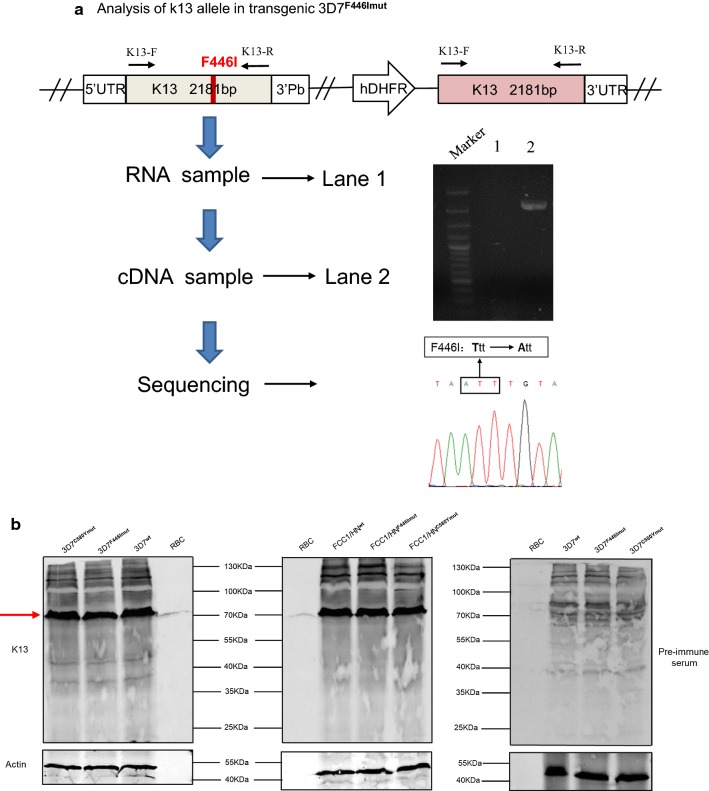
Verification of only *k13* with the F446I mutation expressed. **a** Analysis of expression of both the wild-type and mutant k13 alleles from the 3D7^F446Imut^ parasite. The gel blot shows the K13 gene amplified from the RNA extracted from the transgenic parasite (lane 1, no-RT control) and the cDNA (lane 2); sequence analysis shows all the 12 clones have the F446I mutant alleles. **b** Western blot analysis of *Pf*K13 expression in the transgenic parasite lines and their parent strains

As shown in Fig. 1c, the transgenic parasite with the F446I mutation has two full-length *k13* coding sequences, the mutant and wild-type *k13* alleles. To exclude the possibility that they are both expressed, we generated the full-length *k13* cDNA from the 3D7^F446I mut^ parasite and subcloned the fragments into pEASY-T1 cloning vector for sequencing. As shown in result no *k13* gene was amplified by PCR in the no-RT controls (Fig. 3a, lane 1), indicating no contaminating of genomic DNA in the sample. 12 clones of the recombinant plasmids containing the *k13* gene were sequenced across the F446I mutation. Sequencing data showed that all the 12 clones showed the F446I mutant alleles (none of them are wild-type), indicating that only the mutant allele is expressed.

To investigate whether the integration of the mutant alleles affected the expression of *k13*, the *k13* expression of each transgenic parasite clone were determined with western blot. As shown in Fig. 3b, the expression levels of *k13* in both 3D7^C580Y mut^ and 3D7^F446I mut^ clones were similar to those in the 3D7^wt^ line. Similar expression levels of *k13* were detected in FCC1/HN^wt^ and the transgenic parasite lines FCC1/HN^C580Y mut^ and FCC1/HN^F446I mut^.

### Ring-stage survival of the transgenic parasites with the F446I mutation

**Fig. 4:**
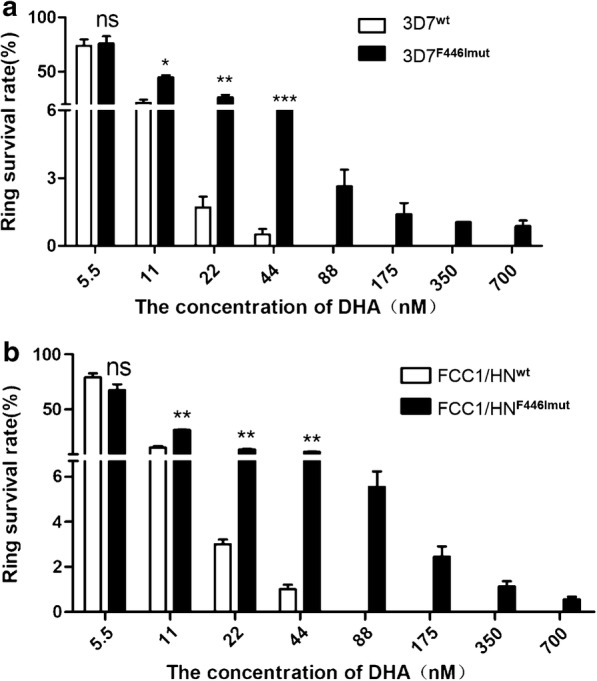
Ring survival of transgenic and parent parasites exposed to elevated concentrations of DHA. **a** Comparison of the survival of 3D7^wt^ and 3D7^F446I mut^ parasites. **b** Comparison of the survival of FCC1/HN^wt^ and FCC1/HN^F446I mut^ parasites

RSA_0–3 h_ was performed to evaluate the transgenic parasite lines expressing F446I and C580Y mutants of *k13* for their susceptibility to DHA. First, ring survival rates of both transgenic parasites and their parent lines were measured when parasites were exposed to elevated concentrations of DHA. As shown in Fig. 4a, at the lowest concentration of DHA (i.e., 5.5 nM), no significant differences in the ring survival rates of the 3D7^wt^ and 3D7^F446I mut^ parasite lines were observed (p > 0.05), while at 11, 22, and 44 nM DHA, the ring survival rates of 3D7^F446I mut^ were significantly higher than that of 3D7^wt^ (p < 0.05, 0.01, and 0.001, respectively). At the concentrations beyond 44 nM, i.e., from 88 to 700 nM, no parasite was observed in the 3D7^wt^ parasite line, and different ring survival rates were detected in the 3D7^F446I mut^ line. A similar result was obtained for the FCC1/HN^F446I mut^ line when compared with its parent parasite strain (Fig. 4b).

Then 2 DHA concentrations (i.e., 20 and 700 nM) from the above-mentioned experiment were selected to further measure the ring survival rates of the F446I and C580Y mutant parasite lines. No parasite of the wild type strain of 3D7^wt^ could survive (survival rate: 0) when 700 nM DHA was used, while the ring survival rates for the transgenic parasite lines of both 3D7^F446I mut^ and 3D7^C580Y^ ^mut^ were similar at 0.73 ± 0.10% and 0.91 ± 0.11%, respectively (Table 1). However, when 20 nM DHA was used, both 3D7^F446I mut^ and 3D7^C580Y mut^ transgenic parasite lines showed high ring-survival rates at 26.0 ± 1.48% and 33.08 ± 1.20%, respectively, whereas the 3D7^wt^ parasites showed a significantly lower survival rate at 3.15 ± 0.13% (p < 0.05). Similar results were obtained after measuring the ring survival rates of FCC1/HN ^F446I^ ^mut^ and FCC1/HN ^C580Y mut^ lines when compared with the FCC1/HN^wt^ strain (Table 1). F08-27 isolate of *P. falciparum* carrying the C580Y mutation collected from China to Myanmar border showed high RSA survival rates [11] and is used as a positive control in this study. The survival rate of the F08-27 isolate was 8.27 ± 0.63% and 65.8 ± 4.06% when the isolate was treated with 700 and 20 nM DHA, respectively.

**Table 1:** The survival rates of transgenic parasite lines with either F446I or C580Y mutation and their parental strains (mean ± SD)

Strains/lines	RSA_0–3 h_ at DHA 700 nM (%)	RSA_0–3 h_ at DHA 20 nM (%)
3D7^wt^	0	3.15 ± 0.13
3D7^C580Ymut^	0.91 ± 0.11	33.08 ± 1.20
3D7^F446Imut^	0.73 ± 0.10	26.0 ± 1.48
Fcc1/HN^wt^	0	2.86 ± 0.44
Fcc1/HN^C580Ymut^	0.63 ± 0.07	17.5 ± 0.80
Fcc1/HN^F446Imut^	0.53 ± 0.06	13.5 ± 0.41
F08-27	8.27 ± 0.63	65.8 ± 4.06

Each assay was repeated for three times independently, with each group setting three technical repetitions

**Fig. 5:**
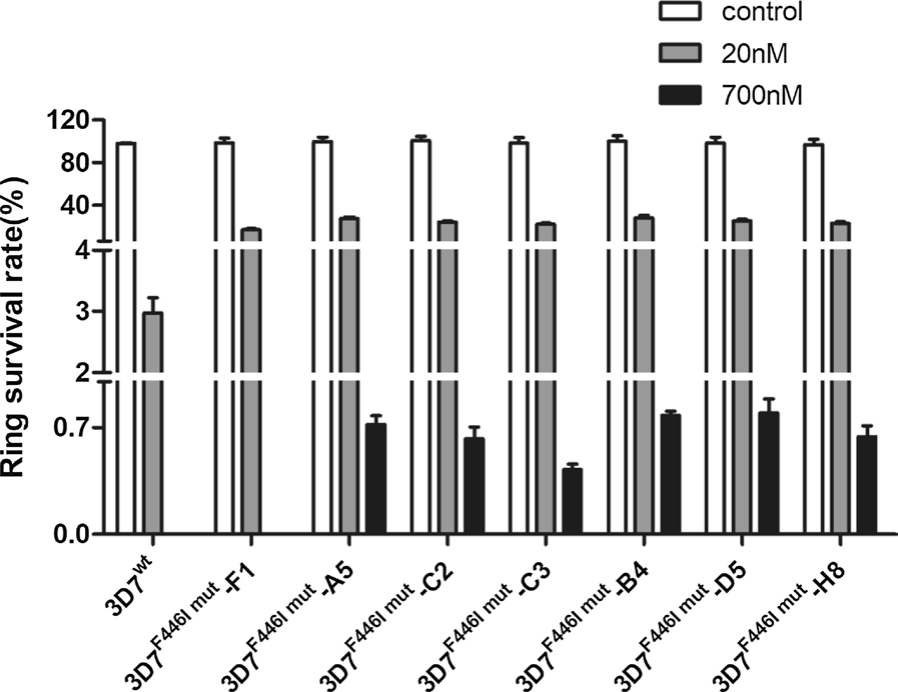
Ring survival of individual clones of 3D7^F446I mut^ transgenic parasites. The survival rate of 3D7 compared with its different F446I mutated sub-cloning under 20 and 700 nM DHA

For generating transgenic 3D7 parasites with the F446I mutation, 7 clones were obtained by sub-cloning the WR99210-resistant parasite by limiting dilution. Then these clones were sequenced to confirm the presence of F446I. All the clones showed increased levels of ring survival from 17.6 to 29.5% at 20 nM DHA when compared with 3.15% survival of 3D7^wt^ (Fig. 5). Interestingly, the survival rate of one clone, 3D7^F446I mut^-F1, expressing the F446I mutant allele was 0 at 700 nM DHA, which is the same as the wild type of the strain. The survival rate was also lower than that of the other clones (17.6%) at 20 nM DHA. The sequencing of the *Pf*K13 gene of the 3D7^F446I mut^-F1 clone showed an additional mutation at nucleotide position 1156 from G to T, resulting in a change in amino acid residue from valine to leucine at position 386 (data not show). It is rare for multisite mutation to occur in *k13* in field isolates. Further investigation is required to understand whether more than 1 mutation in *k13* affects the ART susceptibility of the parasite.

## Discussion

ART resistance is evaluated in vivo by delayed parasite clearance and in vitro by high ring survival rates with RSA_0–3 h_ [8]. The resistant phenotypes are strongly associated with a single mutation after amino acid position 440 in *k13* [7, 10]. Some *k13* mutations, such as C580Y, R539T, Y493H, and I543T, have been reported to be associated with the resistance of parasites to ART [9], and they are highly prevalent in the border areas of Thai/Cambodia and Thai/Myanmar but rare north of Southeast Asia, including north of Myanmar and China–Myanmar border region [7, 19, 20]. Interestingly, north of Southeast Asia, the most prevalent mutant allele of *k13* is F446I. In previous study, the result showed that the prevalence of this mutation is 32.7% in the China–Myanmar border area and this mutation was not detected in Thai/Cambodia and Thai/Myanmar border regions [11]. From 2009 to 2012, Huang et al. [15] conducted a therapeutic efficacy study of artesunate in the China–Myanmar border region and showed that the single mutation of F446I with a prevalence of 36.5% was significantly associated with prolonged parasite half-life and parasitaemia on day 3 after treatment. However, several other studies have found no correlation between this mutation and prolonged parasite clearance time [7, 21]; however, the reason may be the small sample sizes. A previous study found that 2 of 3 parasites with this mutation showed a parasite clearance time < 5 h [22]. A recent study on the therapeutic efficacy of artemether–lumefantrine conducted in the northeast region of India also found no correlation of this mutation with delayed parasite clearance [23].

In previous study, the ring survival rate of 27 isolates with F446I mutation were measured by using RSA_0–3 h_. Of them, only 6 had a high ring survival rate (> P_95_ value) that was defined as the association with delayed parasite clearance in vivo, and the ring survival rates of the other isolates were below the P_95_ value. The statistical analysis indicated that the F446I mutant was not associated with high ring-stage survival. Thus, there is much debate on whether the F446I mutation is associated with ART resistance. In this study, F446I mutation in *k13* was introduced to the ART-sensitive 3D7 and FCC1/HN strains, and the resulting transgenic parasite lines FCC1/HN ^F446I mut^ and 3D7 ^F446I^ ^mut^ showed increased ring survival rates at both 20 and 700 nM DHA concentrations. Moreover, the survival rates of the transgenic parasites with the F446I mutation were similar to those of the parasites with the C580Y mutation demonstrated to be correlated with ART resistance. Thus, the result suggested the F446I mutation may be associated with ART resistance.

The C580Y mutation in *k13* is a primary marker for ART resistance and the most prevalent mutation in western Cambodia where the resistant parasite was first reported. Several transgenic parasite clones have been generated in which the C580Y mutation was introduced into the *k13* gene of various parasite isolates by using the CRISPR–Cas9 technology or zinc-finger nucleases [10, 13]. These transgenic clones showed elevated ring-stage survival when compared with their parent parasites; however, the ring survival rates of these transgenic parasite clones were variable. The 2 transgenic clones expressing *k13* C580Y showed survival rates of 11–15%, while another transgenic FCB parasite clone expressing the C580Y allele showed a survival rate of 1.9% when compared with the survival rate of 0.3% in the parent FCB isolate. We generated both FCC1/HN^C580Y mut^ and 3D7^C580Y mut^ transgenic lines that showed 0.63 ± 0.07% and 0.91 ± 0.11% RSA_0–3 h_ survival at 700 nM DHA concentration, whereas the survival rates of their parent parasites were 0%. Interestingly, when the DHA concentration reduced to 20 nM, both transgenic lines showed 17.5 ± 0.80% and 33.08 ± 1.20% survival rates, whereas their parent parasites showed only 2.86 ± 0.44% and 3.15 ± 0.13% survival rates, respectively. It could be concluded that the introduction of both C580Y and F446I mutations in *k13* of 2 distinct laboratory parasite strains resulted in increased ring survival indicating that, similar to the C580Y substitution, F446I may play role in parasite response to ART.

## Conclusions

In summary, the F446I mutation in *k13* is the most prevalent allele at the China–Myanmar border and north of Myanmar. In this study, this mutation is associated with the increased ring survival rates by genetically introducing the mutation into the *k13* gene of distinct ART-sensitive laboratory parasite strains, suggesting that the F446I mutation be used as a molecular marker for monitoring ART-resistant parasites and highlighting the importance of surveillance of F446I mutants for resistant parasites.

## Abbreviations

*k13*: *P. falciparum* K13 propeller gene
ART: artemisinin
RSA_0–3 h_: ring-stage survival assay of 0–3 h
DHA: dihydroartemisinin
